# Optimized production and characterization of endo-β-mannanase by *Aspergillus niger* for generation of prebiotic mannooligosaccharides from guar gum

**DOI:** 10.1038/s41598-024-63803-4

**Published:** 2024-06-18

**Authors:** Suresh Nath, Naveen Kango

**Affiliations:** Department of Microbiology, Dr. Harisingh Gour Vishwavidyalaya (A Central University), Sagar, Madhya Pradesh India

**Keywords:** β-mannanase, Guar gum, Partially hydrolyzed guar gum, *A. niger*, Prebiotics, Probiotics, Biochemistry, Biotechnology, Microbiology

## Abstract

Optimized production of *Aspergillus niger* ATCC 26011 endo-β-mannanase (*ManAn*) on copra meal resulted in 2.46-fold increase (10,028 U/gds). Purified *ManAn* (47 kDa) showed high affinity towards guar gum (GG) as compared to konjac gum and locust bean gum with K_m_ 2.67, 3.25 and 4.07 mg/mL, respectively. *ManAn* efficiently hydrolyzed GG and liberated mannooligosaccharides (MOS). Changes occurring in the rheological and compositional aspects of GG studied using Differential scanning calorimetry (DSC), Thermal gravimetric analysis (TGA) and X-ray diffraction (XRD) revealed increased thermal stability and crystallinity of the partially hydrolyzed guar gum (PHGG). Parametric optimization of the time and temperature dependent hydrolysis of GG (1% w/v) with 100 U/mL of *ManAn* at 60 °C and pH: 5.0 resulted in 12.126 mg/mL of mannotetraose (M4) in 5 min. Enhanced growth of probiotics Lactobacilli and production of short chain fatty acids (SCFA) that inhibited enteropathogens, confirmed the prebiotic potential of PHGG and M4.

## Introduction

Guar or cluster bean (*Cyamopsis tetragonolobus*) is an annual legume predominantly grown in the semiarid areas of south Asia. Although, grown in other parts of the world also, Rajasthan, Gujarat and Kutch regions of India are the main producers of the cluster bean, accounting for 80% of the world's total production and ~ 406,513 MT of Guar gum worth USD 617.14 million was exported during 2022–2023 (https://apeda.gov.in).

Guar gum is the galactomannan of the seed endosperm of guar and has been explored for a variety of applications^1^. This versatile biopolymer finds numerous applications ranging from oil recovery to preparation of nanocomposites and absorbents for bioremediation^2^. This biocompatible galactomannan has also been used for the generation of prebiotic mannooligosaccharides (MOS)^3^. Mannans, such as, locust bean gum (LBG), konjac gum (KG) and guar gum (GG) are being evaluated as a suitable source for prebiotic MOS and among these, GG is understood to be compatible for the consumption by humans and pets^4,5^.

Microbial β-mannanases find important applications in the biorefinery, food, textile, paper, and pulp industries^6^. Endo-β-mannanases sourced from fungi have been indicated in the production of MOS from guar gum^7,8^. Among these, endo-β-mannanases from Aspergilli such as *A. terreus*, *A*. *quadrilineatus, A. oryzae* and *A. tubingensis* have been used to produce MOS from various mannans^9–12^. *A. niger* is generally regarded as safe (GRAS) and is well-known as a prolific producer of food grade hydrolytic enzymes^13^. Cost incurred in the enzyme production can be minimized by employing agro-industrial wastes as substrates. In case of mannanase production, mannan-rich agro-wastes, palm kernel cake (PKC) and copra meal (CM) have been utilized as substrates in order to make the process cost-effective. Such initiatives also help resolve issues regarding indiscriminate disposal or dumping of mannan-rich agro-wastes.

In the present study, we have demonstrated the optimized production, purification and characterization of an endo-β-mannanase (*ManAn*) from *A. niger* ATCC 26011 using an agro-waste, copra meal. Production of mannotetraose (M4) from GG using *ManAn* was optimized by response surface methodology, MOS were purified by gel-filtration and visualized using Fluorescence assisted carbohydrate electrophoresis (FACE). The prebiotic potential of PHGG and MOS was demonstrated in terms of growth promotion of probiotics and inhibition of pathogens.

## Materials and methods

### Chemicals

Copra meal, a residue of copra oil industry, was procured from Parker Biotech Pvt. Ltd, Chennai, India. Guar gum (GG), locust bean gum (LBG), 4-nitrophenyl (pNP) substrates, pNP α-d-galactopyranoside (pNP Gal), pNP β-d-mannopyranoside (pNP M), pNP β-d-glucopyranoside (pNPG), 3,5 di-nitrosalicylic acid (DNS), diethylaminoethyl (DEAE) cellulose-52, monopotassium 7-amino-1,3-naphthalenedisulfonate monohydrate (ANDS), sodium cyanoborohydride, Biogel-P2, deuterium oxide (D_2_O) and silica gel 60 F_254_ TLC plates were obtained from Sigma Chemical Co., USA. Various growth media ingredients were obtained from HiMedia Laboratories, Mumbai, India. MOS standards: mannose (M1), mannobiose (M2), mannotriose (M3) and mannotetraose (M4) were obtained from Megazyme, Ireland. Fructooligosaccharides (FOS) were purchased from Unique Biotech, Hyderabad, India. HPLC grade water (AS077) obtained from HiMedia Laboratories, had ammonium, cadmium, copper, cobalt, iron and lead less than 0.00005% and non-volatile substances less than 0.0003%.

### Microorganisms

*Myceliophthora thermophila* NFCCI 3725, *Malbranchea cinnamomea* NFCCI 3724, *Aspergillus niger* ATCC 26011, *Aspergillus oryzae* MTCC 1846, *Lactobacillus delbrueckii* NCIM 2025, *Lactobacillus acidophilus* NCIM 5306, *Lactobacillus rhamnosus* MTCC 5957, *Staphylococcus aureus* MTCC 96 and *Candida albicans* MTCC 227 were procured from various culture collections. *Aspergillus quadrilineatus* RSNK-1 and *Aspergillus tubingensis* NKBP-55 are native isolates and are maintained in the Enzyme Technology and Molecular Catalysis (ETMC) Laboratory, Dept. of Microbiology, Dr. Harisingh Gour Vishwavidyalaya, Sagar (MP), India.

### Production of β-mannanase

#### Screening of fungal strains for β-mannanase production

Mycelial discs (4) of the three-day old fungal cultures were inoculated on 5 g of copra Meal (CM) moistened with distilled water (1:3) and incubated for 4 days at 45 °C (thermophilic fungi) or 28 °C (mesophilic fungi). The enzyme was harvested by stirring the fermented CM in 50 ml citrate buffer (50 mM; pH 5.0) for 1 h at 4 °C and the slurry was filtered through Whatman filter paper no.1^7^. β-mannanase and other accessory mannanolytic enzyme activities were estimated in the crude cell-free culture filtrate as described earlier^14^. Briefly, LBG solution (0.9 ml, 0.5% w/v in 50 mM citrate buffer, pH 5.0) and appropriately diluted enzyme (0.1 ml) were mixed and incubated at 50 °C for 10 min. Afterwards, DNS reagent (1.5 mL) was added, tubes were boiled for 5 min and the absorbance was measured at 540 nm. One unit of β-mannanase was defined as the amount of the enzyme that liberated 1 μmol of mannose per min under the assay conditions. α-galactosidase, β-glucosidase and β-mannosidase activities were determined utilizing pNPGal, pNPG and pNPM, respectively^10^. pNP substrates (1 mM) were made to react with suitably diluted enzymes samples for 10 min at 50 °C. Subsequently, 1 ml of sodium carbonate solution (10% w/v) was added as the stopping reagent and the absorbance was measured at 415 nm. The amount of enzyme required to release 1 μmol of pNP from the respective substrate per min was referred as one unit^15^.

#### Optimization of β-mannanase production from *A. niger* ATCC 26011

Initially, production of β-mannanase from *A. niger* ATCC 26011 was optimized using one-variable at a time (OVAT) approach. Mannanase production was studied by varying one variable, while keeping the others constant in solid state fermentation (SSF). The effect of pH (4.0–10.0), moisture (distilled water: 0 ml to 30 ml), temperature (10 °C to 50 °C) and time (1–5 days) on the production of β-mannanase from *A. niger* was studied*.* After ascertaining most influential parameters by OVAT, central composite design (CCD) of independent variables, A-moisture (CM: water ratio ~ 1:2–1:4) and B-pH (6.0–8.0), was used to study the interaction of these parameters for the optimized production of β-mannanase. Experimental design and analysis were done using Design Expert 11.0. As per CCD, 13 experiments based on a 5-level (–α, –1, 0, + 1, + α), 2-factor design with axial point (4), factorial point (4) and replicate trials of central point (5) were conducted. β-mannanase activity (U/gds) was calculated as the response of the designed experiment and second order polynomial equation was used for the prediction of optimum value based on response surface method (RSM)^11^.

### Purification and characterization of *A. niger* ATCC 26011 endo-β-mannanase

Total protein was precipitated using chilled ethanol (1:3) and was collected by centrifugation at 10,000*g* for 10 min at 4 °C. The protein was resuspended in citrate buffer (pH 5.0) and was dialyzed through a 30 kDa membrane against the same buffer overnight at 4 °C^9^. Anion exchange chromatography was conducted at room temperature using DEAE cellulose-52 in a gravity column and the fractions were eluted using a linear gradient of NaCl (100–1000 mM) in Tris–HCl (pH 8.4). Protein in fractions was measured at 280 nm and *ManAn* activity was assayed by DNS method.

The molecular weight (MW) of *ManAn* was ascertained using SDS-PAGE and zymography using 12% native resolving gel containing 0.05% (w/v) LBG. Culture filtrate, protein dialysate and the DEAE-cellulose fraction having maximum *ManAn* activity were resolved on the gel at 100 V for 2 h at 4 °C. After electrophoresis, part of the gel containing protein marker (Biorad, USA) was stained with Coomassie brilliant blue R-250, while the other part was incubated at 50 °C for 60 s. After incubation, it was stained with Congo red (0.1% w/v) and de-stained using NaCl solution (1 M) for 10 min to visualize zone of LBG hydrolysis formed due to β-mannanase activity^10^. The temperature optima of *ManAn* was obtained by performing enzyme activity in the range of 30–100 °C, while its thermostability was determined by pre-incubating it at 50, 60, 70, 80 and 90 °C for 120 min. Optimum pH of *ManAn* was determined using LBG (0.5% w/v) dissolved in various buffers, *viz*. sodium-citrate (pH 4.0-5.0), disodium hydrogen phthalate-sodium dihydrogen orthophosphate (pH 6.0), Tris-HCl (pH 7.0-9.0) and glycine-sodium hydroxide (pH 10.0). pH stability was determined by pre-incubating *ManAn* at pH 4.0-8.0 up to 120 min at room temperature. Lineweaver–Burk (LB) plots were prepared by incubating *ManAn* with various concentrations of LBG, GG and KG and the kinetic parameters K_m_, V_max_ and K_cat_ were determined^16^.

### Optimization of MOS generation and purification

For this, GG (1% w/v) was incubated with *ManAn* (100 U/mL) at 50 °C for varying time intervals (5–300 min). Henceforth, the effect of temperature was examined by varying incubation temperature (30–100 °C). The reaction was stopped by boiling the *ManAn*-GG reaction mix for 5 min and the samples were analyzed by TLC^17,18^. After drawing inference from the above experiment, RSM was used to enhance the production of mannotetraose (M4). Two independent variables, time and temperature were optimized by CCD using Design Expert 11.0 software. Total 13 experiments with 4 trials of factorial design, 4 trials of axial point and 5 replicate trials of central point were conducted and the response pattern (M4 concentration) to evaluate the optimum combination of factors. The responses were measured using Waters HPLC system (Empower-2 Software) and the concentration of various MOS, including M4 was calculated in mg/mL.

After hydrolysis of GG using *ManAn* under optimized conditions various MOS were purified using Biogel-P2. The column was prepared by activating the polyacrylamide beads in HPLC grade water overnight at 4 °C, autoclaving and degassing prior to use. PHGG (2 mL) was added to the column and was allowed to pass through with the help of gravitational pull. The elution of the sample was done with HPLC grade water at a flow rate of 0.5 mL/min. After 40 min of adding the sample on the size-exclusion column, fractions were collected up to 2 h and were analyzed using HPLC and FACE^18^.

### Rheological and thermal properties of PHGG

MW of GG and PHGG was estimated by rheometric analysis (MCR502S, Anton Paar GmbH, Austria) and was calculated according to the Huggins equation, i.e.

$$\upeta_{{{\text{red}}}} = \, \left[ \upeta \right] \, + {\text{ K}}\left[ \upeta \right]^{{2}} {\text{C}}$$

where, ɳ_red_ is reduced viscosity, K is Huggin’s constant (3.8 × 10^4^),

C is the concentration of GG or PHGG and the intercept of the plot represents the intrinsic viscosity (ɳ).

The viscosity average molar mass (M_v_) of GG or PHGG was calculated using the Mark-Houwink-Sakurada (MHS) equation^19^, i.e.

$$\left[ \upeta \right] \, = {\text{ K}}_{[\upeta ]} \times {\text{ M}}_{{\text{v}}}{^{\upalpha }}$$

where, K_[η]_ and α are constants for the given solvent, polymer and temperature (for the distilled water, K_[η]_ = 4.34 × 10^–4^, α = 0.73).

For further analysis, the GG and PHGG samples were lyophilized for converting them into powder without distorting their internal structure. Moisture-free samples were spread homogenously on conductive adhesive carbon tape and were coated with gold for the analysis. Topology of GG and PHGG was visualized by SEM at 16,000 X under 10 kV continuous scanning voltage (FEI Nova NanoSEM 450). Thermal properties of GG and PHGG were analyzed using differential scanning calorimetry (DSC) and thermal gravimetric analysis (TGA), while changes in crystallinity patterns were studied by X-ray diffraction (XRD) analysis^20^.

### Prebiotic properties of PHGG and purified M4

Three probiotic Lactobacilli, *L. delbrueckii* NCIM 2025, *L. acidophilus* NCIM 5306,

*L. rhamnosus* MTCC 5957 were inoculated (A_600_ ~ 0.6) in M9-minimal medium containing 0.5% (w/v) different carbon source (PHGG, M4, glucose, mannose or FOS). The flasks were incubated under anaerobic condition (AnaeroGas Pack-Jar, HiMedia) at 37 °C. The growth of the probiotic strains (A_600_) was measured at 12 h interval upto 48 h. All the experiments were carried out in triplicates (p-value < 0.05) and the values represent average ± standard deviation (SD).

### Production of SCFA and antimicrobial postbiotics

*S. aureus* MTCC 96 and *C. albicans* MTCC 227 were seeded on nutrient agar (NA) and malt extract-glucose-peptone-yeast extract agar (MGYPA), respectively and cell-free culture filtrate (100 µl) obtained after 48 h of growth of probiotics on different carbon sources was poured in the 6 mm well. The plates containing *S. aureus* and *C. albicans* were incubated at 37 °C and 28 °C, respectively and were examined for zone of inhibition after 24 h. Culture filtrates obtained after the growth of probiotics on M9-minimal medium and M9 supplemented with GG were used as controls.

The culture filtrates obtained after the growth of probiotics on PHGG containing medium were analyzed for the presence of postbiotic metabolites by NMR spectroscopy. These were passed through 0.22 μm filter and mixed with 10% D_2_O for ^1^H and ^13^C NMR spectroscopy at 125.7 MHz resolution frequency at an angle of 30°. The samples were measured using JEOL 500 MHz NMR spectrometer and Mnova 15.0.1 software was used for preparing the graphs. Chemical Book (https://www.chemicalbook.com/SpectrumEN_64-19-13CNMR.htm) was used for analysing the NMR spectra (ppm) for confirming the production of postbiotics.

## Results and discussion

### Screening of fungal strains for β-mannanase production

Two thermophilic and four mesophilic fungi were explored for the production of β-mannanase and associated mannanolytic enzymes (Table 1). Thermophilic *M. thermophila* NFCCI 3725 and *M. cinnamomea* NFCCI 3724 were grown at 45 °C, while other fungi were cultured at 28 °C for 4 days on copra meal with water as moistening agent (1:3) in SSF. Results clearly indicated that *A. niger* ATCC 26011 produced high titres of β-mannanase (4064 U/gds), α-galactosidase (233 U/gds), β-glucosidase (68 U/gds) and β-mannosidase (24 U/gds). Sornlake et al.^21^ used wheat bran, rice bran, locust bean gum, copra meal and palm kernel meal and found that copra meal was the best carbon source for β-mannanase production with high mannanase titres (1837.5 U/gds) by *A. niger* BCC 4525. Alsarrani^22^ have reported that *A. niger* produced the highest extracellular mannanase activity (2.90 U/ml), followed by *Aspergillus flavus* (2.54 U/ml) and *Aspergillus ochraceous* (2.16 U/ml), however, no β-mannosidase activity was seen after 6 days of incubation under submerged conditions. Previously, *M. cinnamomea* NFCCI 3724 has been shown to produce 669 U/gds of β-mannanase^23^. Out of 36 fungi isolated from PKC, *A. niger* IBRL F16.A4 was reported to be the best for β-mannanase production (17.82 ± 0.05 U/mg)^24^. When *A. niger* USM F4 was grown in a small tray system with PKC, a low-cost agricultural waste widely available in Malaysia, high mannanase titre (918.68 U/g) was obtained^25^. Olaniyi^26^ isolated fungi from Ilaje lake, Nigeria and β-mannanase of *A. niger* 4B1 had highest specific activity (2.59 U/mg) in contrast to other isolates, *Penicillum italicum*, *Fusarium solani*, *A. flavus*, *Rhizopus stolonifer*, *A. fumigatus*, *R. japonicus* and *C. albicans*. Based on the maximum titres produced by *A. niger* ATCC 26011 in SSF on copra meal, it was selected for the optimized production of β-mannanase.

**Table 1:** Production of mannanase and other accessory enzymes by some fungi.

Fungus	β-mannanase (U/gds)	α-galactosidase (U/gds)	β-glucosidase (U/gds)	β-mannosidase (U/gds)
*Myceliophthora thermophila* NFCCI 3725	623.07 ± 13.35	27 ± 3.58	11.06 ± 2.45	26.06 ± 2.06
*Malbranchea cinnamomea* NFCCI 3724	269.23 ± 6.22	47.05 ± 4.82	18 ± 2.73	22 ± 2.09
*Aspergillus niger* ATCC 26011	4064 ± 20.16	233.08 ± 7.45	68.09 ± 3.74	24 ± 2.63
*Aspergillus quadrilineatus* RSNK-1	1060 ± 17.72	86.43 ± 7.55	23.06 ± 5.62	10 ± 1.03
*Aspergillus oryzae* MTCC 1846	434 ± 11.54	34 ± 4.35	27 ± 5.66	11 ± 1.62
*Aspergillus tubingensis* NKBP-55	1023 ± 13.05	54 ± 6.27	72 ± 7.09	28 ± 2.1

All the strains were cultured on Copra meal moistened with water (1:3 w/v) in SSF and the enzyme yields (U/gds) represent average of three replicates ± standard deviation.

### Optimized production of β-mannanase from *A. niger* ATCC 26011

**Figure 1:**
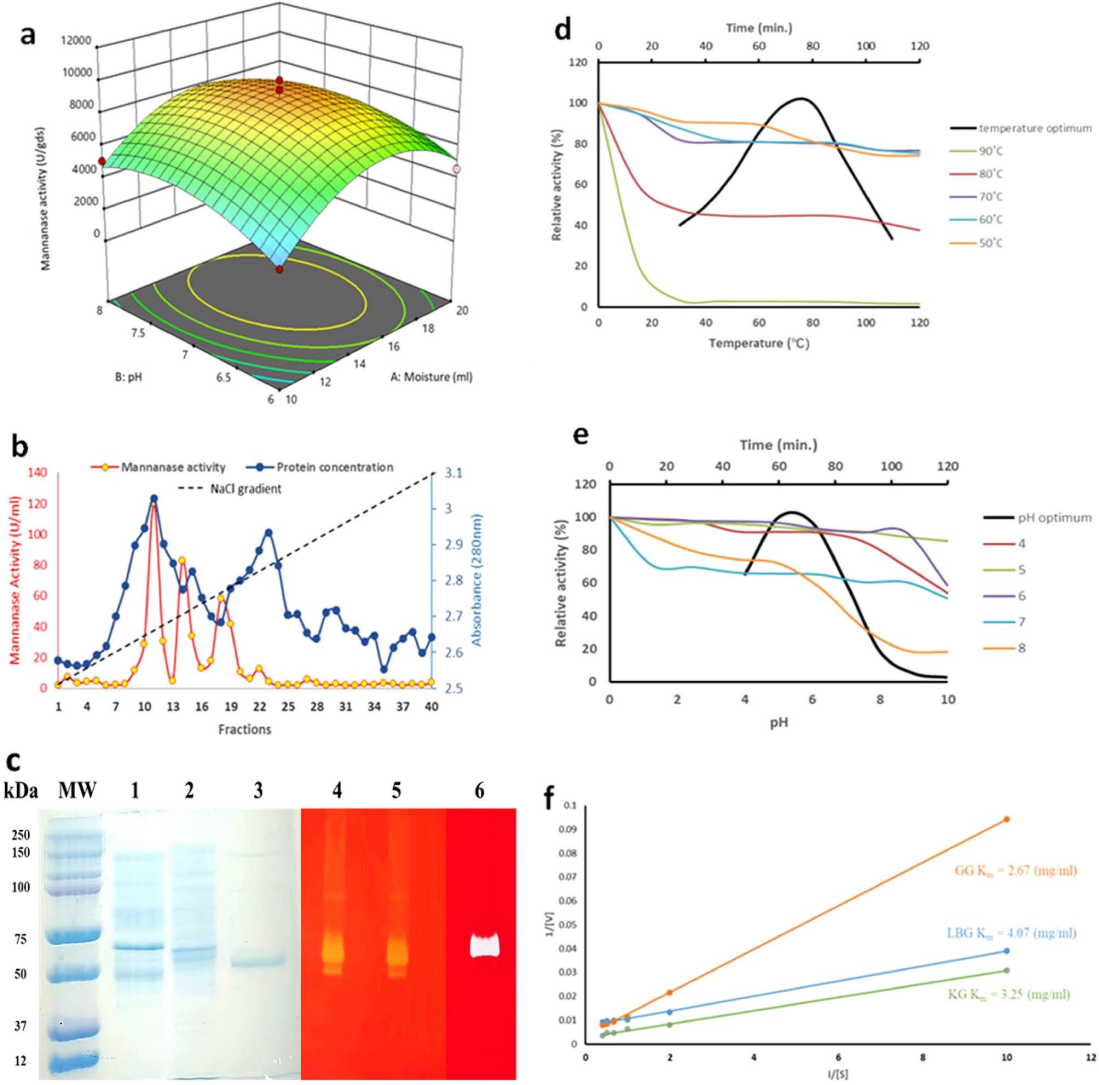
Optimized production and characterization of *A. niger* ATCC 26011 endo-β-mannanase. (**a**) RSM showing interaction of pH and moisture content. Agro-waste copra meal was used as substrate in SSF. (**b**) Purification profile of *ManAn* using DEAE cellulose-52. (**c**) SDS-PAGE showing protein profile and zymogram (47 kDa and 40 kDa) (MW: mol. wt. marker, Lane 1: crude enzyme, Lane 2: Dialyzed protein precipitate, Lane 3: purified *ManAn* (47 kDa), Lanes 4–6: Zymogram of Lanes 1–3 (**d**) temperature optimum and thermal stability (**e**) pH optimum and pH stability (**f**) Lineweaver–Burk plots showing affinity of purified *ManAn* towards GG, KG and LBG.

*Aspergillus niger* ATCC 26011 produced 4064 U/gds of β-mannanase under un-optimized conditions on CM in SSF. As per the inferences drawn from OVAT experiment (Supplementary Fig. 1), pH and moisture content were used as variables for statistical optimization. Pasanen et al.^27^ have observed moisture to be the most influential parameter for enzyme production under SSF conditions. After optimizing these parametric values by CCD, 2.46-fold increase in mannanase titre (10,028.9 U/gds) was achieved (Fig. 1a). The confirmation of quadratic model was caried out under optimum conditions i.e., moisture content (CM: water 1:3) and pH (7.0) and the model F-value of 28.11 and p-value less than 0.05 confirmed that the model was significant and moreover, the F-value of 0.51 implied that the lack of fit was not significant. Previously, 1.37-fold increase in β-mannanase production by *A. niger* (1495 nkat/ml to 2063 nKat/ml) was achieved after medium optimization using RSM^28^, while Yin et al*.*^29^ showed 2.18-fold increase in β-mannanase production by *A. niger* SN-09 (256.5 U/gds to 561.3 U/gds) under optimized conditions. Our findings suggest CM to be a suitable low-value substrate for the production of β-mannanase by *A. niger* in SSF*.* Mulimani and Naganagouda^30^ also found CM to be a better inducer of β-mannanase for *A. niger* among various carbon sources (LBG, GG, glucose, mannose, galactose and xylose). High mannan content (mannose: galactose ratio in copra mannan ~ 14:1)^31^ and nutritional value of the CM supports the fungus to produce mannanase efficiently. Sornlake et al.^21^ used CM moistened with basal media for production of β-mannanase from *A. niger* BCC4525 and 1837 U/gds was produced in SSF at 30 °C in 3 days. Mohamad et al.^28^ optimized β-mannanase production from *A. niger* ATCC 20114 under submerged conditions using basal medium supplemented with GG or LBG (30 °C, 150 rpm, 11 days) and observed 1495 nkat/ml and 1148 nkat/ml of mannanase yield, respectively. The β-mannanase production by *A. niger* in the present study was significantly higher than the previous reports (Table 2).

**Table 2:** A comparative report on production strategies of β-Mannanase from *A. niger.*

*A. niger* strain	Production conditions				Mannanase activity	Reference
	Media components	Temp (°C)	Shaking (rpm)	Time (days)		
*A. niger* NCH-189	Mandel–Reese’s medium with carbon source substituted with defatted copra meal	30	120	3	28 U/ml	^32^
*A. niger* gr	2% LBG in minimal media	37	180	7	40 U/ml	^33^
*A. niger* gr	2% (LBG, GG or defatted copra meal)	40	120	5	40 U/ml	^16^
*A. niger* FTCC 5003	Palm kernel cake moistened (60%) with Mandel–Reese’s medium in an aerated column bioreactor	32.5	–	7	2117.89 U/gds	^34^
*A. niger*	LBG in growth media	30	120	6	2.9 U/ml	^22^
*A. niger* ATCC 20114	Basal medium supplemented with GG or LBG	30	150	11	GG, 1495 nkat/ml LBG, 1148 nkat/ml	^28^
*A. niger* BCC 4525	Copra meal moistened with basal media	30	–	3	1837 U/gds	^21^
*A. niger* ATCC 26011	Copra meal moistened with water (7pH)	28	–	4	10,029 U/gds	This study

### Purification and characterization of mannanase from *A. niger* (*ManAn*)

Various precipitation methods for recovery of β-mannanase and total protein from *A. niger* culture filtrate were evaluated and ethanol (1:3) was found to be the most suitable (Supplementary Fig. 2). Total protein was precipitated using chilled ethanol and dialysed through a 30 kDa cut-off membrane. About 53.38% mannanase was recovered from the crude extract. The dialyzed precipitate was loaded on the anionic exchange column (DEAE cellulose-52) and the protein was eluted by linear NaCl gradient. Fractions collected at 300 mM, 400 mM and 500 mM NaCl concentration had the maximum *ManAn* activity as well as protein content (Fig. 1b). *ManAn* was purified to apparent homogeneity after passing the dialysate through DEAEcellulose-52 with 16.5% recovery (Table 3). Zymogram of the culture filtrate and dialysed precipitate showed the presence of two mannanases, *viz*. of 40 and 47 kDa, while the fraction eluted with 300 mM NaCl showed the presence of a single band of 47 kDa (Fig. 1c). Magengelele et al.^35^ have reported a 47.8 kDa β-mannanase from *A. niger* ATCC 10864, while Wu et al.^36^ have found a 42 kDa β-mannanase of *A. niger* E-30 grown on wheat bran supplemented with LBG.

**Table 3:** Purification profile of *ManAn* cultivated on CM in SSF.

Purification profile	Total Activity (U)	Protein (mg)	Sp. activity (U/mg)	Purification fold	Yield recovery (%)
Crude	1253.56	62.44	20.07	1	100
Dialysis (30 kDa membrane)	669.24	7.24	92.43	4.6	53.38
DEAE cellulose-52	206.92	0.4	517.3	5.59	16.50

*ManAn* had optimum activity at 80 °C and retained 44.63% residual activity at this temperature after 1 h incubation, whereas it showed considerable stability at 70 °C and retained 76.73% relative activity after 2 h pre-treatment (Fig. 1d). Similarly, Huang et al.^37^ showed that β-mannanase from *A. niger* BK01 had optimum activity at 80 °C and was stable in the range of 50–70 °C. The purified β-mannanase retained 50% activity after 6 h treatment at 55 °C^16^. *ManAn* exhibited optimum activity at pH 5.0 and retained 85.63% residual activity at this pH after 2 h of incubation, indicating tolerance for acidic pH. With the increase in pH, the activity of β-mannanase was reduced and only 18.12% residual activity remained after 2 h of incubation at pH 8.0 (Fig. 1e). Wu et al.^36^ reported β-mannanase of *A. niger* LW-1 to be highly stable at acidic pH while, Naganagouda et al.^16^ observed β-mannanase of *A. niger* to be optimally active at pH 5.5 and reduced to less than 50% at pH 8.

The kinetic parameters of the purified *ManAn* with different mannans are summarized in Table 4. The substrate affinity of *ManAn* towards different mannans was in the order of GG > KG > LBG, with K_m_ 2.67, 3.25 and 4.07 mg/ml, respectively (Fig. 1f). This revealed that the *ManAn* had more affinity towards the GG, a galactomannan as compared to KG, a glucomannan. Similarly, Bien-Cuong et al.^38^ also reported that purified mannanase of *A. niger* BK01 had higher affinity towards galactomannan (GG > LBG > KG). Mannanase of *A. niger* gr also showed higher affinity towards GG. Mafa and Malgas^44^ stated that GH26 mannanases have high affinity towards GG than other mannans and are prominently used in the production of MOS. Endo-β-mannanase of *A. niger* belongs to GH26 CAZy family of glycosyl hydrolases. In the present study also, *A. niger* ATCC 26011 mannanase showed higher affinity towards GG. A comparison of methods for purifying mannanase from *A. niger* and its characteristics including affinity towards various mannans is presented in Table 5.

**Table 4:** Kinetic parameters of *ManAn*.

Kinetic parameters	Mannans		
	LBG	GG	KG
V_max_ (U/ml)	131.57 ± 2.1	294.11 ± 2.7	357.14 ± 3.3
K_m_ (mg/ml)	4.07 ± 0.7	2.67 ± 0.2	3.25 ± 0.5
K_cat_ (s^-1^)	5.48 ± 0.4	12.25 ± 0.5	14.88 ± 0.7

Values represent average of three replicates ± standard deviation (V_max_: maximum velocity; K_m_: Michaelis–Menten constant; K_cat_: turnover number; LBG: locust bean gum; GG: guar gum; KG: konjac gum).

**Table 5:** A comparative account of purification and properties of *A. niger* mannanase (SEC: size exclusion chromatography; AEC: anion exclusion chromatography; AC: affinity chromatography; LBG: locust bean gum; GG: guar gum; CG: carob gum; CM: copra meal; KG: konjac gum).

Purification method	Mannanase characteristics							References
	Specific activity (U/mg)	Temp optima (℃)	pH	K_m_ (mg/ml)	V_max_ (U/mg)	MW (kDa)	Major products	
Superdex (SEC)	2570	80	4.5	2 (LBG) 0.6 (KG) 2.2 (CG)	373 (LBG) 243 (KG) 330 (CG)	53	M2, M3	^38^
DEAE-Sephacel (AEC)	65.06	55	5.5	0.11 (LBG) 0.28 (GG) 0.33 (CM)	14.13 (LBG) 11.23 (GG) 7.2 (CM)	66	–	^16^
Sephadex G-75 (SEC)	–	70	3.5	1.1 (LBG)	266.7 (LBG)	52	–	^39^
Sephadex G-75 (SEC)	–	50	4.8	3.68 (LBG)	1067.5 (LBG)	42	M3	^36^
Superdex G-75 (SEC)	1139.2	45	5.0	26.50 (LBG) 2.87 (GG) 23.50 (KG)	5000 (LBG) 769.20 (GG) 1250 (KG)	45	MOS (DP 2–6)	^40^
Ni–NTA (AC)	–	80	4.5	3.5 (LBG)	374.8 (LBG)	43	M2, M3	^41^
DEAE-Sepharose (AEC)	802	70	5.5	2.16 (LBG)	1533.1 (LBG)	–	M2	^42^
Sephacryl S100 HR (SEC)	–	45	5.0	–	–	66	MOS (DP-2–6)	^43^
DEAEcellulose-52 (AEC)	517.3	80	5.0	4.07 (LBG) 2.67 (GG) 3.25 (KG)	131.57 (LBG) 294.11 (GG) 357.14 (KG)	47	M4	This study

### Production optimization and purification of M4 from PHGG

**Figure 2:**
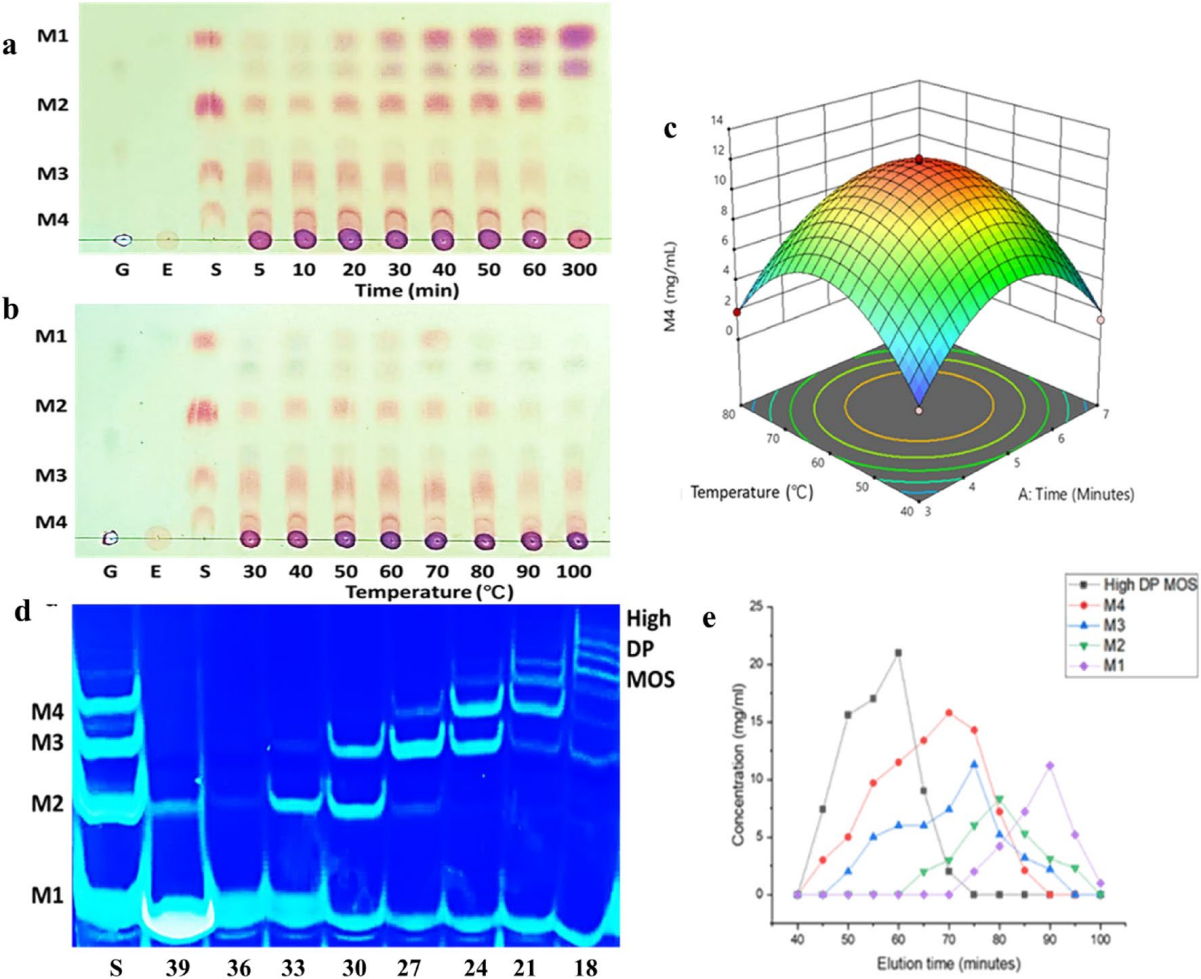
Optimized production of MOS from GG by *ManAn*. (**a**) TLC showing time dependent GG hydrolysis. (**b**) TLC showing temperature dependent GG hydrolysis. (**c**) Parametrically optimized production of MOS using RSM. (**d**) MOS fractionation using Bio-gel P2 as visualized by FACE (S: standards and fractions 18–39). (**e**) Elution profile of MOS through Bio-gel P2.

TLC analysis showed production of MOS from GG, wherein maximum production with minimum amount of mannose was noticed after 5 min of incubation and with the increase in time more mannose was produced (Fig. 2a). This indicated the need of controlled enzymatic reaction so that higher concentration of MOS is obtained without further degradation of GG to mannose. PHGG production at various temperatures was also visualized on TLC, showing maximum MOS production at 60 °C (Fig. 2b). Chen et al.^45^ also optimized time, temperature, pH and enzyme to substrate ratio for the production of gluco-mannooligosaccharides from KG using RSM and observed temperature and incubation period to be significant in hydrolysis of KG. Thus, these two variables (time and temperature) were parametrically optimized using RSM and a ten-fold increase in M4 production from 1.118 to 12.126 mg/mL was observed at 60 °C in 5 min hydrolysis of GG (1%) with *ManAn* (100 U/mL) (Fig. 2c). Gluco-mannooligosaccharides production was significantly enhanced (0.283 mg/mL to 3.768 mg/mL) after optimizing hydrolysis conditions (41 °C, pH 7.1, incubation time 3.4 h and E/S 0.49) ^45^. Yilmazer et al.^46^ optimized the production of MOS from GG using a recombinant *Aspergillus sojae* β-mannanase and observed 233 ppm of M4, under the optimized parameters i.e., GG concentration 0.75%, 40 °C and incubation time 35 min in a stirred tank bioreactor. The MOS production method used in the present study lead to production of 12.126 mg/mL of M4 in 5 min treatment of 1% (w/v) GG with *ManAn* (100U/mL) at 60 °C.

**Figure 3:**
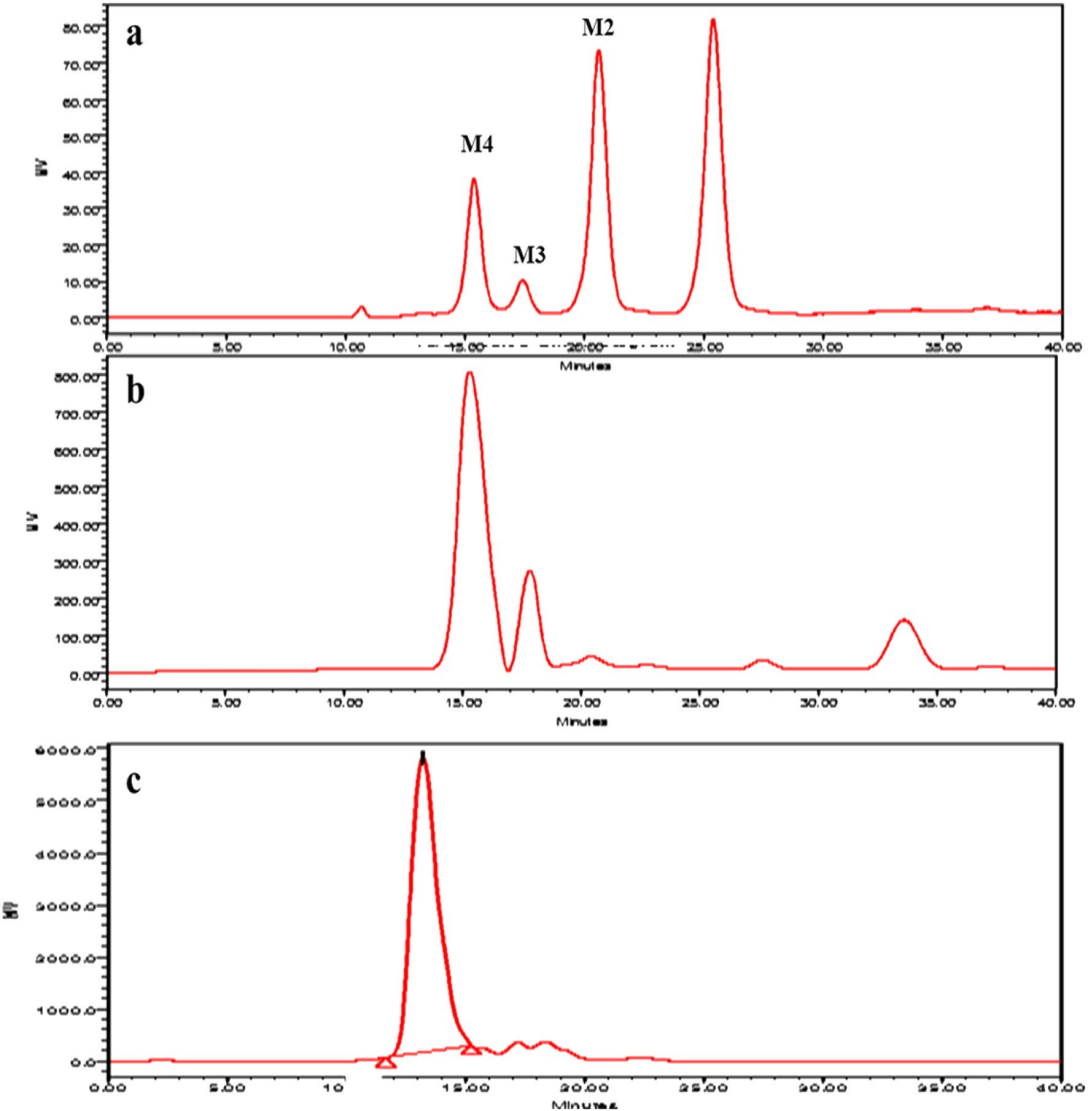
HPLC showing MOS generation by *ManAn* from GG (**a**) MOS standards (**b**) Guar gum hydrolysate showing presence of MOS (c) Biogel-P2 fraction containing purified mannotetraose (M4).

La Rosa et al.^47^ described that low DP MOS (M4, M3, M2) are more efficient and possess better prebiotic potential. Thus, M4 was purified from PHGG mixture and was evaluated for prebiotic potential. M4 was purified from the PHGG mixture in a single-step using size-exclusion Biogel-P2 column. FACE analysis was performed to visualize the resulting sugars from the mannan hydrolysate which showed the presence of high DP MOS till 18th fraction, while low DP MOS (M2-M4) eluted out after 21st fraction (Fig. 2d). Fraction 24 and 27 showed the presence of M4 and M3, respectively, while M2 and M1 can be seen in fraction 33 and 39, respectively. M4 (~ 13 mg/mL) and M3 (~~ 10 mg/mL) were eluted during 55–65 min and 65–75 min, respectively (Fig. 2e). Nopvichai et al.^48^ also purified MOS using Biogel-P2 column and visualized the purified fractions from M4 to M7 on TLC. Similarly, Jana and Kango^18^ purified M3 (1.26 mg/mL) and M2 (0.68 mg/mL) from GG-MOS using Biogel-P2 column. Quantitative analysis by HPLC confirmed the presence of M4 as the major end-product of GG hydrolysis. After purification, 95.16 mg/g of purified M4 was obtained, showing 86% product recovery (Fig. 3). MOS obtained after hydrolysis of defatted CM were subjected to Biogel-P2 in an FPLC system and M2 (40%) and M3 (18%) were recovered^49^. Recently, GG pre-treatment with citric acid and its enzymatic hydrolysis resulted in 61.8% MOS. MOS was purified using ion exchange resins (D-301 anion resin and D-001 cation resin) to 90% purity and 4.57 g of solid MOS was recovered from 10 g of GG after spray drying^50^.

### Rheological and thermal properties of PHGG

**Figure 4:**
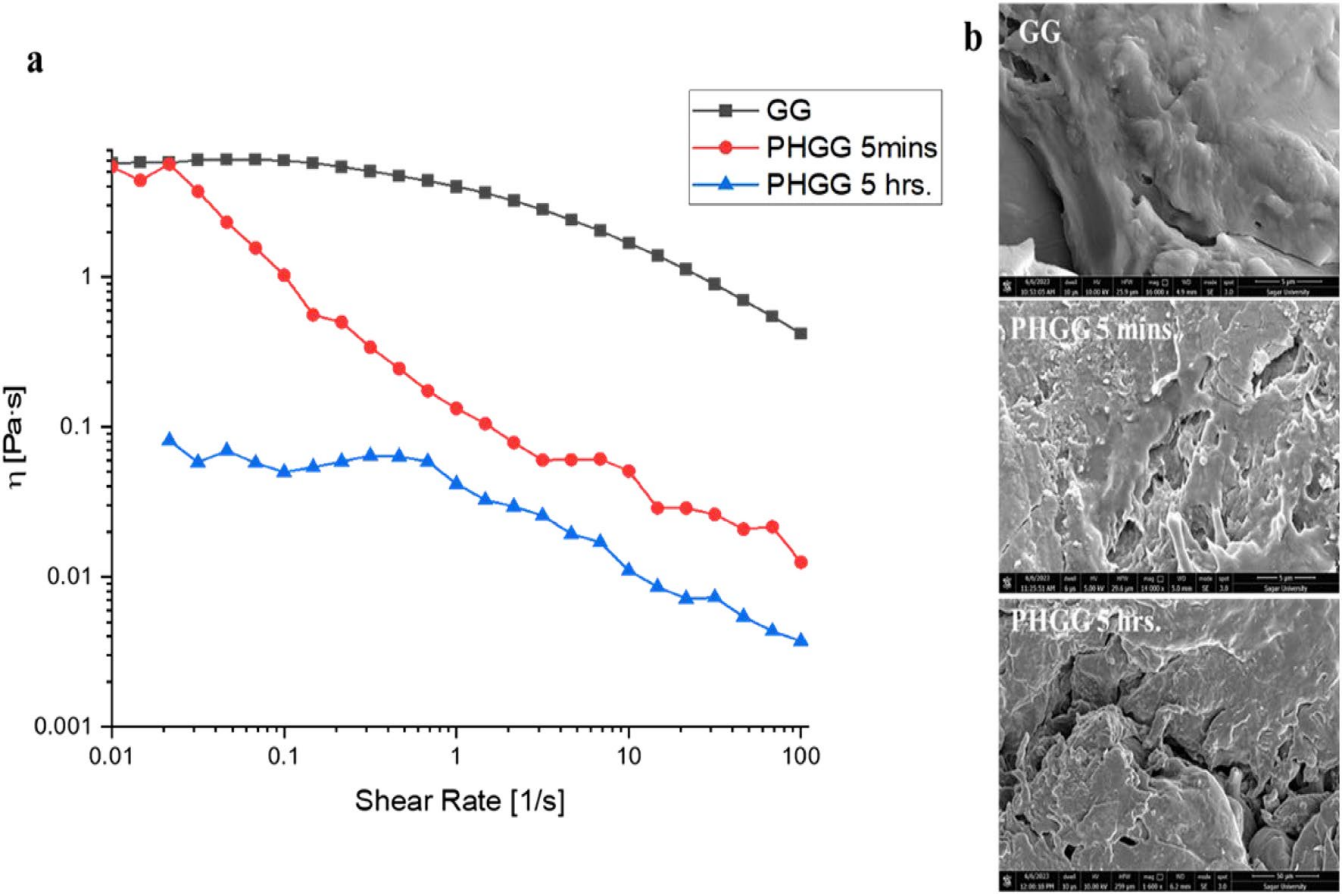
Rheological properties of PHGG. (**a**) Viscometric analysis and molecular weight determination of hydrolysate using *ManAn*. (**b**) Scanning electron micrographs showing morphological alterations in guar gum upon treatment with *ManAn.*

Rheometric analysis of GG and PHGG suggested that mannanase treatment leads to significant decrease in the viscosity of GG (Fig. 4a). MHS equation was used to calculate the MW of untreated GG (76,226.16 Da), PHGG after 5 min incubation (50,948.16 Da) and PHGG after 5 h incubation (10,286.16 Da), suggesting that the decrease in the MW is due to the breakdown of the glycosidic bonds of GG due to hydrolytic action of *ManAn*. The decrease in the viscosity makes it more palatable and suitable for application in food and feed industry. Based on the viscosity analysis, Mudgil et al.^20^ reported the MW of GG to be 889,742.06 Da and after 4 h of enzymatic hydrolysis the MW of PHGG was reduced to 7936.5 Da. Depending upon the source, preparation process and the storage conditions, the MW of GG can be from 50,000 to 8,000,000 Da^18^.

Topographical depiction by SEM images indicated the morphological changes brought about by the mannanase treatment in the structure of GG and PHGG (Fig. 4b). The surface of GG was smooth and flat, whereas roughness, breakage and depressions were seen in PHGG, confirming the cleavage of polymer to produce short chain oligosaccharides. Recently, Hussain et al.^51^ also visualized GG and PHGG using SEM and reported that GG existed in granular form, whereas PHGG showed amorphous and porous characteristics.

**Figure 5:**
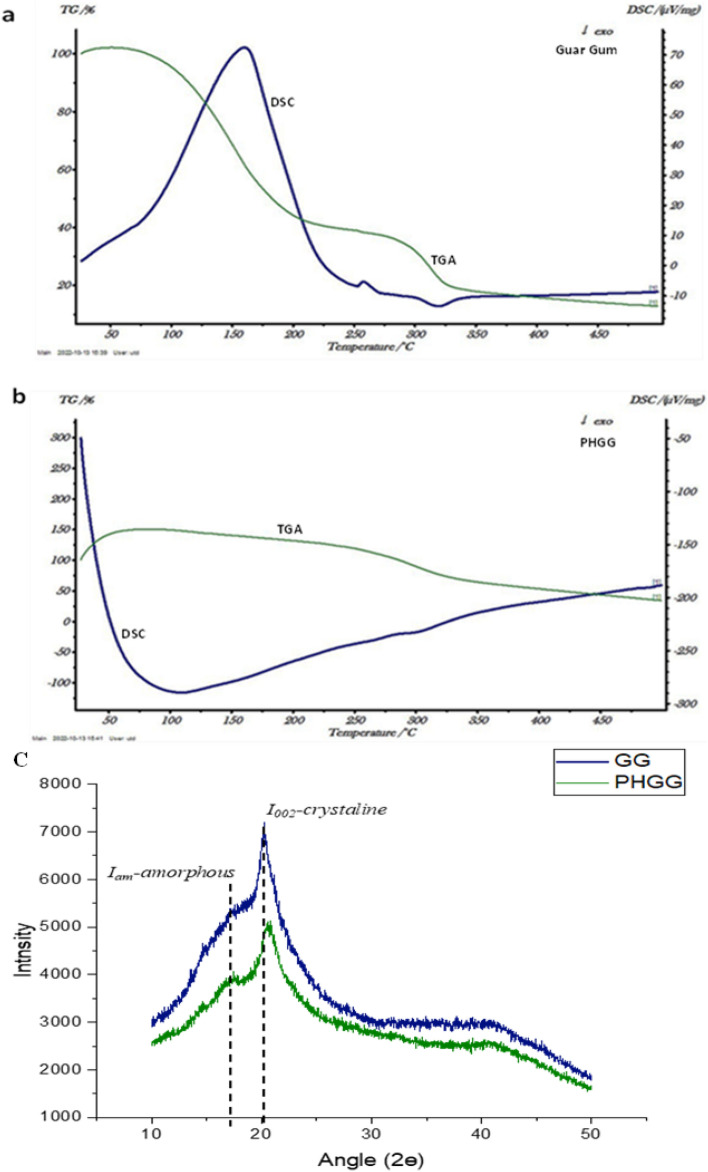
Changes in thermal behavior of guar gum after hydrolysis with *ManAn* (**a**) GG and (**b**) PHGG (**c**) XRD showing the crystallinity index of GG and PHGG.

DSC curve of GG showed an endothermic peak at 150 °C indicating the early decomposition and cleavage of galactose and mannose units from GG backbone (Fig. 5a). Another small endothermic peak at 250 °C represents the initiation of combustion of polymer whereas, an exothermic peak at 350 °C represents the oxidative decomposition of GG, vaporization and elimination of volatile products. Mudgil et al.^20^ reported endothermic peaks of GG at 253 °C and 296 °C, and exothermic peak was detected at 317 °C. The endothermic peaks for GG were detected at 245 °C and 313 °C, and the exothermic peak was detected at 368 °C^52^. An exothermic peak at 100 °C of PHGG represents immediate combustion of PHGG (Fig. 5b). TGA displays the thermal behaviour of the polymer. The thermal stability of GG decreased with the increase in temperature (Fig. 5a) whereas, PHGG was more thermostable (Fig. 5b). This shows increased thermal stability of PHGG than GG at higher temperature, making it more suitable for food industry applications like baking, frying and sterilization processes. TGA profiles of residual mass confirmed increased stability of PHGG at higher range of temperature as compared to GG^53^.

XRD patterns of GG and PHGG shown in (Fig. 5c) confirm their amorphous nature. The crystalline regions of GG and PHGG were seen at the angle (2ɵ) 20.2. There is no change in the XRD pattern upon enzymatic treatment of GG, but the lower intensity of PHGG represents the breakage of polymer into smaller molecules. Mudgil et al.^20^ also reported crystalline regions of GG and PHGG at the angle (2ɵ) 20.2 and 72.5 and also witnessed no major change in the XRD pattern of GG and PHGG.

### Prebiotic properties of PHGG and purified M4

**Figure 6:**
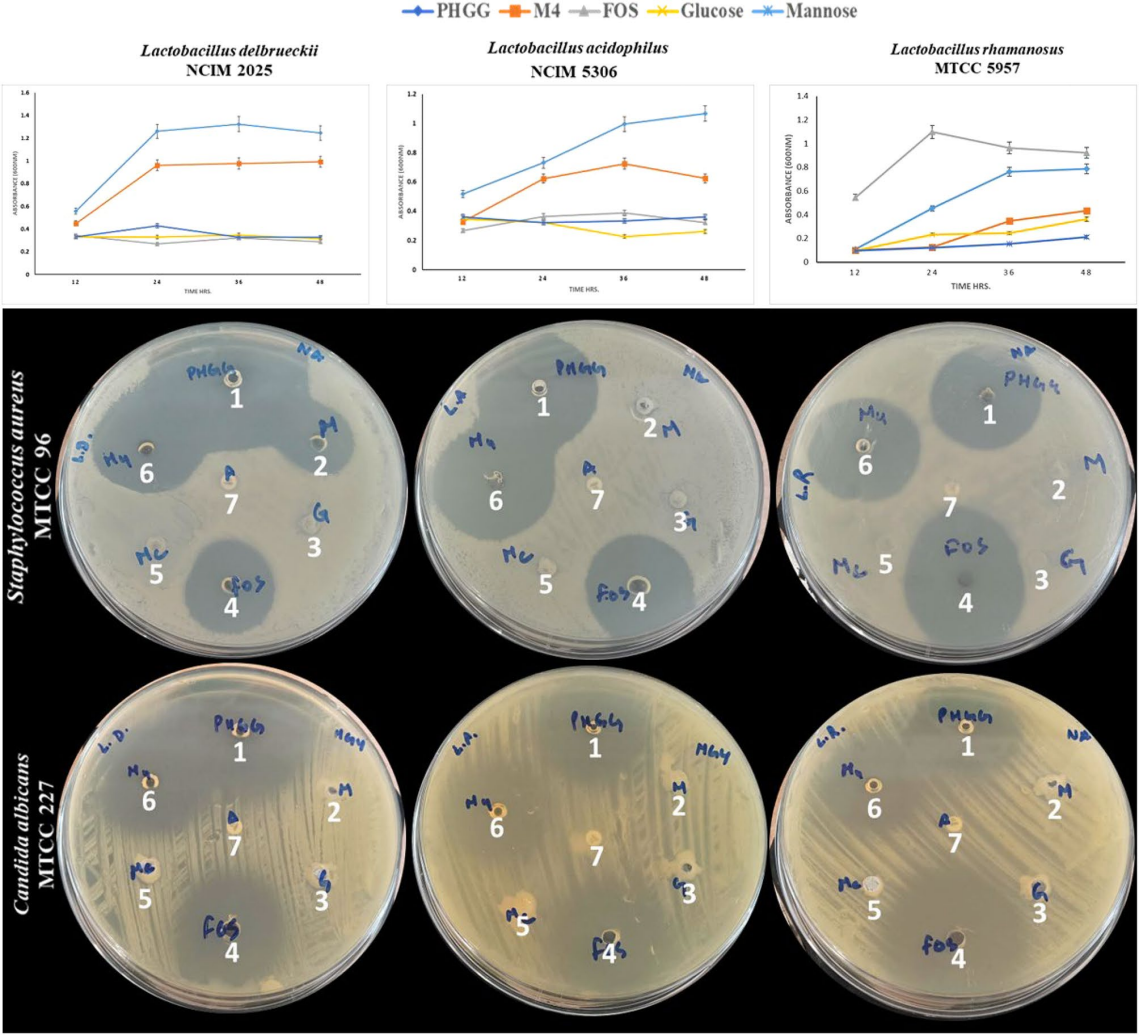
Growth of probiotic (**a**) *L. delbrueckii* NCIM 2025, (**b**) *L. acidophilus* NCIM 5306, (**c**) *L. rhamnosus* MTCC 5957 in M9-minimal medium containing PHGG, M4, FOS, glucose and mannose as sole carbon source and the effect of postbiotic metabolites on pathogenic *S. aureus* MTCC 96 and *C. albicans* MTCC 227 (1-PHGG, 2-Mannose, 3-Glucose, 4-FOS, 5-medium control, 6-M4 and 7-Guar gum control).

Among the three Lactobacilli grown on different carbon sources, *L. delbrueckii* NCIM 2025 and *L. acidophilus* NCIM 5306 showed better growth on PHGG and M4 in comparison to other carbon sources, while *L. rhamnosus* MTCC 5957 grew well on FOS (Fig. 6). Thus, the probiotic Lactobacilli selectively utilized PHGG and M4 and accordingly a synbiotic preparation containing PHGG or M4 may be prepared. Jana et al.^18^ reported that *L. delbrueckii* NCIM 2025 growth was enhanced with the hydrolysates of various galacto- and gluco-mannans in comparison to glucose. Mary et al.^54^ also suggested that DP2 and DP3 (GG-β-MOS) are utilized to different extents by *Lactobacillus* strains. MOS derived from spent coffee grounds promoted the growth of LAB in vitro and *L. casei* culture filtrate inhibited the growth of *S. paratyphi*^55^. PHGG supplementation significantly increased the production of SCFA and relative abundance of human beneficial probiotic bacteria in a human intestinal in vitro fermentation model^56^.

### Production of SCFA and antimicrobial postbiotics

Culture filtrates produced after the utilization of PHGG, M4, FOS, glucose and mannose were analyzed for the presence of antimicrobial activity against pathogens. Culture filtrate of *L. delbrueckii* NCIM 2025, *L. acidophilus* NCIM 5306 and *L. rhamnosus* MTCC 5957, when grown on PHGG, M4 and FOS as carbon sources, formed distinct zones of inhibition against enteropathogenic *S. aureus* MTCC 96 and *C. albicans* MTCC 227, whereas no zone of inhibition was seen for mono sugars like, glucose and mannose (Fig. 6). *L. rhamnosus* MTCC 5957 showed larger zones of inhibition for both the pathogens when grown on FOS in comparison to PHGG and M4,  whereas *L. delbrueckii* NCIM 2025 and *L. acidophilus* NCIM 5306 showed superior zones of inhibition with PHGG and M4. This study suggested that *L. delbrueckii* NCIM 2025 and *L. acidophilus* NCIM 5306 with PHGG or M4 are effective in inhibiting enteric pathogen, *S. aureus* and *C. albicans*. Mary et al.^54^ reported that DP2/DP3 and GG-β-MOS mixture inhibited the growth of enteropathogens in monoculture and co-culture fermentations. Choi et al.^57^ observed inhibition of various food-borne pathogens by the cell-free supernatant of various probiotic bacteria grown on De Man, Rogosa, and Sharpe (MRS) broth. Bai et al.^58^ studied the production of antibacterial peptides from *Bacillus subtilis* and *Lactobacillus buchneri* grown on silage. The bioactive principles involved in antimicrobial activity and the mechanism thereof need further investigations.

**Figure 7:**
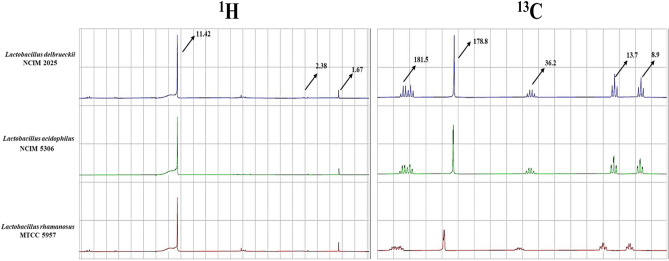
Characterization of postbiotics using ^1^H and ^13^C NMR spectra of the culture filtrates of the *Lactobacillus* spp. grown on PHGG. The peaks show the production of short chain fatty acids (SCFA).

Key SCFAs in complicated samples can be found using nuclear magnetic resonance 1H-NMR with little sample preparation^59^. The NMR spectra of culture filtrates of *L. delbrueckii* NCIM 2025, *L. acidophilus* NCIM 5306 and *L. rhamnosus* MTCC 5957 grown on PHGG as sole carbon source were analyzed for the presence of postbiotics (https://www.chemicalbook.com/SpectrumEN_64-19-13CNMR.htm). The peaks in ^1^H and ^13^C NMR spectra coincide with the peaks which are available in the database for acetic acid, propionic acid and butyric acid (Fig. 7). The NMR spectra, thus, confirmed the production of SCFA after fermentation of PHGG by the probiotic *Lactobacillus* spp. Wang et al.^60^ studied the selective utilization of sucrose by *Staphylococcus epidermidis,* evaluated the metabolites produced using NMR spectroscopy and confirmed the presence of SCFA after fermentation for its further use against *Propionibacterium acnes*. Müller et al.^61^ analysed SCFAs ratios in the fecal samples of depressed young adults using NMR spectroscopy and found that depressive symptoms are directly related to the acetate levels and indirectly related to butyrate and propionate levels. Tan et al.^62^ suggested G-protein coupled receptors (G-pcr) act as receptors for various SCFA and studies implicate the role of G-pcr in inflammation and phagocytosis, thus could be related to the inhibition of enteropathogens. Moreover, SCFA are known to lower the pH of lumen of the intestine making it unfavourable for the growth of enteropathogens. Recently, Zhang et al.^63^ demonstrated production of MOS from GG (5 g/L) with mannanase (160 U/gds). The MOS had prolific prebiotic potential and supported growth and SCFA production by Lactobacilli which, in turn, decreased abundance of pathogens by 37.80%.

## Conclusion

β-MOS derived from plant mannans are being explored as potential prebiotics. The current work focused on the use of copra meal, a low-value mannan-rich agricultural waste, in the production of MOS generating mannanase from *A. niger*. The mannanase was purified and further employed for the optimized generation of PHGG and M4 from guar gum. The prebiotic potential of PHGG and M4 was confirmed as they supported the growth of probiotic Lactobacilli, and the postbiotics thus produced, inhibited the growth of pathogenic *S. aureus* and *C. albicans*. Therefore, the present study demonstrated a comprehensive approach of MOS generation from guar gum and their prebiotic potential.

## Supplementary Information

Supplementary Figures.

## Data Availability

All data generated or analysed during this study are included in this published article.

## Abbreviations

*ManAn*: Endo-β-mannanase of *Aspergillus niger* ATCC 26011
CM: Copra meal
SSF: Solid state fermentation
OVAT: One variable at a time
RSM: Response surface methodology
CCD: Central composite design
GG: Guar gum
LBG: Locust bean gum
KG: Konjac glucomannan
PHGG: Partially hydrolysed guar gum
MOS: Mannooligosaccharides
FOS: Fructooligosaccharides
M1: Mannose
M2: Mannobiose
M3: Mannotriose
M4: Mannotetraose
MW: Molecular weight
DP: Degree of polymerization
TLC: Thin layer chromatography
HPLC: High performance liquid chromatography
FACE: Fluorescence assisted carbohydrate electrophoresis
SDS-PAGE: Sodium dodecyl sulphate–polyacrylamide gel electrophoresis
SCFA: Short chain fatty acids
pNP: 4-Nitrophenyl
DNS: 3,5 Di-nitrosalicylic acid
DEAE: Diethylamino ethyl
ANDS: Monopotassium 7-amino-1,3-naphthalenedisulfonate monohydrate
NFCCI: National Fungal Culture Collection of India
ATCC: American Type Culture Collection
NCIM: National Collection of Industrial Microorganisms
MTCC: Microbial type culture collection
DSC: Differential scanning calorimetry
TGA: Thermal gravimetric analysis
XRD: X-ray diffraction
SEM: Scanning electron microscope
MHS: Mark-Houwink-Sakurada equation
