# Supplementary material for: Optimized production and characterization of endo-β-mannanase by Aspergillus niger for generation of prebiotic mannooligosaccharides from guar gum
Source: Sci Rep. 2024 Jun 18;14:14015. doi: 10.1038/s41598-024-63803-4 (PMC11637063; doi:10.1038/s41598-024-63803-4)
Supplement: Supplementary file 1 — Supplementary Figures. [file 41598_2024_63803_MOESM1_ESM.docx]

**Supplementary Data**


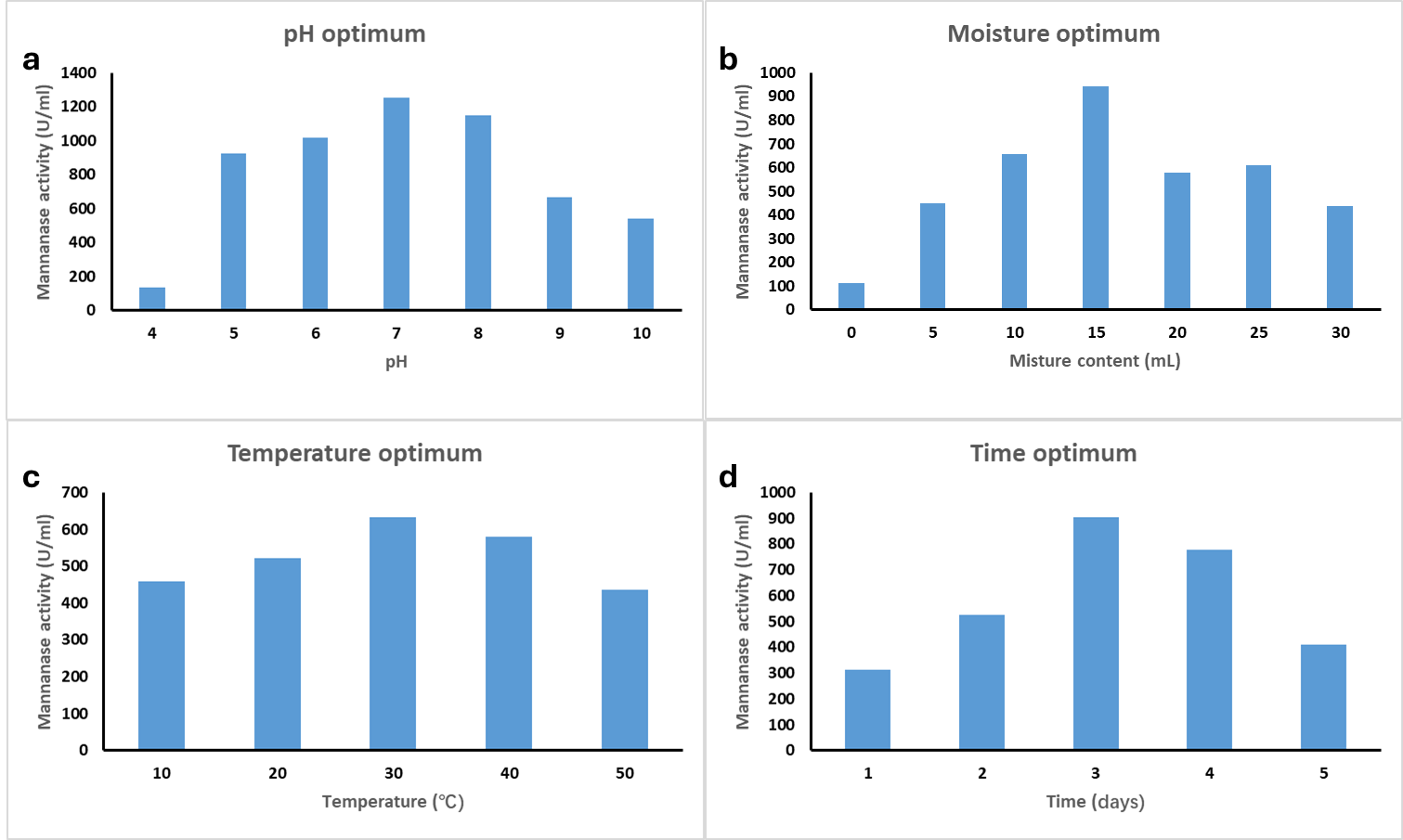


**Supplementary Figure 1:** One variable at a time (OVAT) approach for optimum mannanase production by *A. niger* ATCC 26011 (a) pH; (b) moisture content; (c) temperature; (d) time.

**Supplementary Figure 2:** Recovery of *A. niger* ATCC 26011 endo-β-mannanase after precipitation with various reagents.


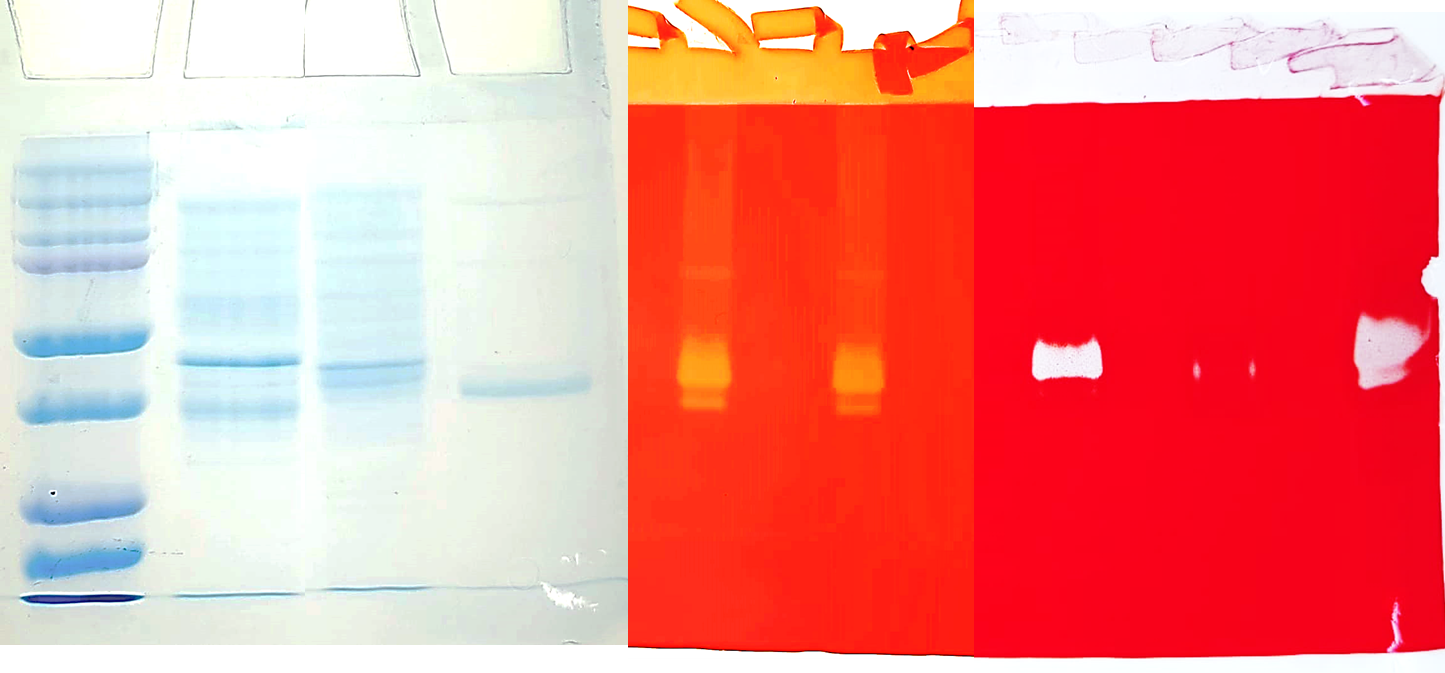


**Supplementary Figure 3:** SDS-PAGE analysis showing protein profile of endo-β-mannanase *A. niger* ATCC 26011 and purified and Zymogram of the fractions.


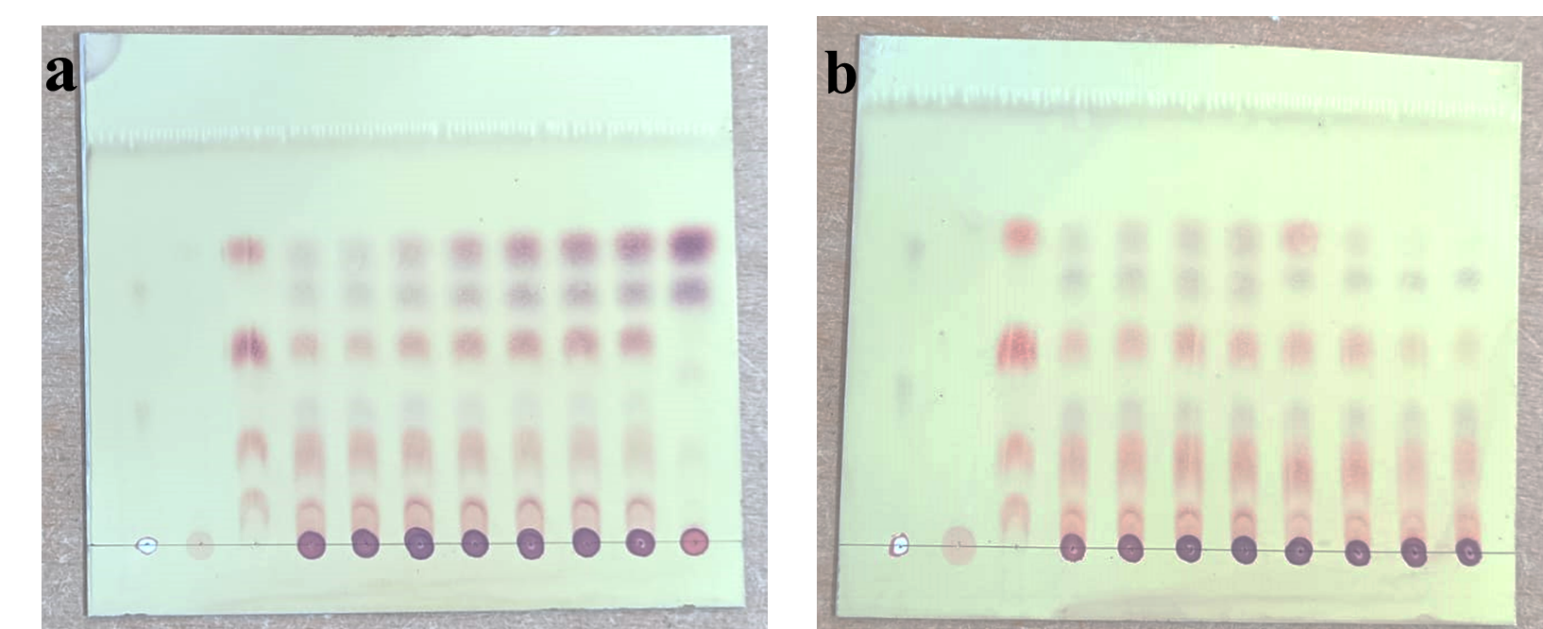


**Supplementary Figure 4:** Thin layer chromatograms of guar gum hydrolysates by mannanase. (a) hydrolysis at different time intervals; (b) hydrolysis at different temperatures.

**
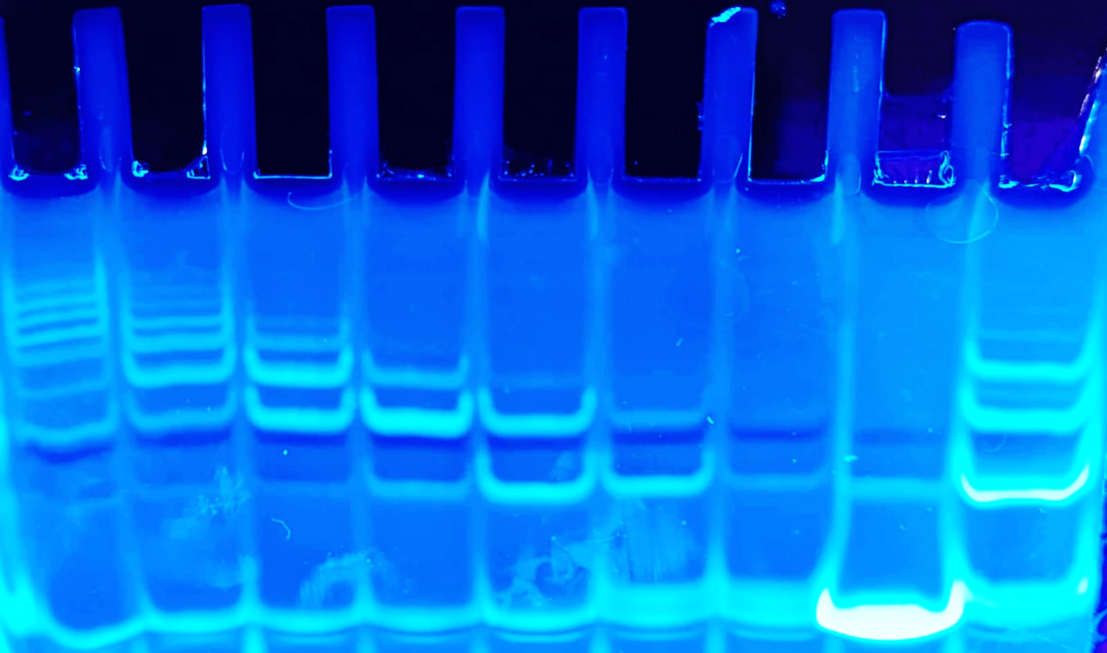
**

**Supplementary Figure 5:** Fluorescence assisted carbohydrate electrophoresis (FACE) showing the purification profile of MOS after passing PHGG mixture through Biogel-P2 size exclusion column.
